# Relationship between Neuroprotective Effects and Structure of Procyanidins

**DOI:** 10.3390/molecules27072308

**Published:** 2022-04-02

**Authors:** Juan Chen, Yixuan Chen, Yangfan Zheng, Jiawen Zhao, Huilin Yu, Jiajin Zhu

**Affiliations:** Department of Food Science and Nutrition, Zhejiang University, Hangzhou 310000, China; chenjuan544676305@163.com (J.C.); chenyixuan2021419@163.com (Y.C.); zhenyangfan2021419@163.com (Y.Z.); 21813083@zju.edu.cn (J.Z.); yuhl0323@126.com (H.Y.)

**Keywords:** procyanidins, neuroprotective, PC12 cells, zebrafish, Nrf2/ARE pathway

## Abstract

This study evaluated the relationship between the neuroprotective effects of procyanidins and their structural characteristics. In vitro, a rat pheochromocytoma cell line (PC12) was exposed to the grape seed-derived procyanidin monomers: catechin (C), epicatechin (EC), and epicatechin gallate (ECG); the procyanidin dimers: procyanidin B1 (B1), procyanidin B2 (B2), procyanidin B3 (B3), procyanidin B4 (B4), procyanidin B1-3-*O*-gallate (B1-G), and procyanidin B2-3-*O*-gallate (B2-G); and the procyanidin trimers: procyanidin C1 (C1) and *N*-acetyl-l-cysteine (NAC) for 24 h. Cells were then incubated with 200 μM H_2_O_2_ for 24 h. In vivo, zebrafish larvae (AB strain) 3 days post-fertilization were incubated with NAC or procyanidins (C, EC, ECG, B1, B2, B3, B4, B1-G, B2-G, C1) in 300 µM H_2_O_2_ for 4 days. Different grape seed procyanidins increased the survival of PC12 cells challenged with H_2_O_2_, improved the movement behavior disorder of zebrafish caused by H_2_O_2_, inhibited the increase of ROS and MDA and the decrease of GSH-Px, CAT, and SOD activities, and up-regulated the Nrf2/ARE pathway. The neuroprotective effects of the procyanidin trimer C1 treatment group were greater than the other treatment groups. These results suggest that the neuroprotective effect of procyanidins is positively correlated with their degree of polymerization.

## 1. Introduction

Neurodegenerative disease refers to disorders caused by chronic, progressive degeneration or deletion of neurons in the human brain [1], mainly characterized by protein accumulation and synaptic loss or dysfunction of neurons in the central nervous system [2]. Because neurons in the central nervous system do not regenerate, damage to these cells cannot be reversed. Consequently, once neurodegenerative diseases occur, the effects are permanent [3]. Therefore, the identification of effective methods for the prevention of neurodegenerative diseases is essential. Although the underlying mechanisms of neurodegenerative disease are not fully understood, there is evidence that oxidative damage caused by excessive production of reactive oxygen species (ROS), free radicals, and damage to the antioxidant defense system play a key role. Therefore, antioxidant supplementation may prevent or alleviate the onset of neurodegenerative disease [4,5,6]. Natural bioactive compounds are generally safe and cause minimal to no side effects. Therefore, identification of antioxidant compounds from natural resources that may aid in the prevention of neurodegenerative diseases is an important research focus.

Procyanidins are natural antioxidants with an antioxidant capacity stronger than that of vitamins C and E [7,8,9]. Previous research from our team has shown that procyanidins exhibit neuroprotective effects [10]. Interestingly, individual procyanidins have distinct effects, which may be due to differences in their structure such as the number of hydroxyl groups, the positioning of the hydroxyl groups, the degree of polymerization, and the type of flavane-3-alcohol unit bond [11]. The bioavailability of procyanidins is also affected by their degree of polymerization [12]. Currently, the effects of procyanidin structure on their neuroprotective effects remains clear.

Grape seed is one of the most abundant sources of procyanidins in nature, and most procyanidin products are derived from grape seed [13,14]. Grape seed procyanidins mainly include type B procyanidins connected by C4–C8 or C4–C6 bonds. Therefore, it is important to study the structure-activity relationship of procyanidins from grape seed. The higher the number of structural units and isomers, the more difficult it is to identify and separate procyanidins for analysis. In addition, due to a lack of effective analysis methods, it is also difficult to isolate pure procyanidin polymers that have a high degree of polymerization [15,16]. In the current study, monomer, dimer, and trimer procyanidins from grape seeds that could be isolated and purified were selected for study of the relationship between the neuroprotective effects of procyanidins and their structural characteristics.

The morphology, structure, and function of the rat pheochromocytoma cell line (PC12) are similar to that of human neuronal cells. Therefore, this is a widely used neuronal cell model [17,18,19,20,21]. Approximately 87% of zebrafish genes are homologous with human disease genes, and the neurobehavioral phenotypes (learning, sleep, and drug addiction) of zebrafish are comparable to human beings [22,23,24,25]. The neurotransmitter system of zebrafish (cholinergic, dopaminergic, and noradrenergic pathways) has been fully documented, and the suitability of this model for the study of nervous system diseases has been confirmed [26,27]. In addition, compared with mice, experiments with zebrafish require a shorter amount of time, which minimizes time and cost [28]. In this study, PC12 cells and the zebrafish model were used to study the relationship between the neuroprotective effects of procyanidins and their structural characteristics.

## 2. Materials and Methods

### 2.1. Chemical Compounds and Reagents

Cell Counting Kit-8 was obtained from Beyotime (Shanghai, China). Catechin (C), epicatechin (EC), epicatechin gallate (ECG), procyanidin B1 (B1), procyanidin B2 (B2), procyanidin B3 (B3), procyanidin B4 (B4), procyanidin B1-3-*O*-gallate (B1-G), procyanidin B2-3-*O*-gallate (B2-G), and procyanidin C1 (C1) were obtained from Caoyuankang Biotechnology. 2′,7′-Dichlorofluorescin diacetate, malondialdehyde (MDA), glutathione peroxidase (GSH-Px), superoxide dismutase (SOD), and catalase (CAT) diagnostic kits were obtained from Solarbio (Beijing, China). Nuclear factor-erythroid 2-related factor 2-siRNA (Nrf2-siRNA), control-siRNA, and Lipofectamine 2000 were obtained from GenePharma (Shanghai, China). The primary antibodies, Nrf2, heme oxygenase 1 (HO-1), NAD(P)H: quinone oxidoreductase 1 (NQO1), Lamin B, and GAPDH, as well as corresponding secondary antibodies, were obtained from Proteintech (Wuhan, China). RNAiso Plus, PrimeScript RT reagent Kit with gDNA Eraser, and SYBR Premix Ex Taq II were purchased from Takara (Takara, Shiga, Japan).

### 2.2. Cell Culture

PC12 cells were obtained from the National Collection of Authenticated Cell Cultures. Cells were maintained in DMEM supplemented with 10% fetal bovine serum and penicillin-streptomycin (100 U/mL; 100 μg/mL) in a humidified atmosphere incubator at 37 °C with 5% CO_2_.

### 2.3. Cell Viability Assay

PC12 cells were incubated with C, EC, ECG, B1, B2, B3, B4, B1-G, B2-G, C1 or *N*-acetyl-l-cysteine (NAC) (20 μM) for 24 h and then incubated with 1 H_2_O_2_ (200 μM) for 24 h. Cell Counting Kit-8 solution (10 μL) was added to each well and incubated for 1 h. The absorbance was measured at 450 nm.

### 2.4. Fish Maintenance

Zebrafish (wild-type AB strain) were maintained in a breeding environment at 28.0 °C, with a light cycle (light/dark) of 10 h/14 h. Fish were fed once every morning and evening. The day before egg retrieval, zebrafish were put into the mating box at a female to male ratio of 1:1. The next morning, the partition plate was removed. After the fish laid eggs, the eggs were collected and put into the incubator (28.5 °C). The ethics certificate approval No. for this study was zju20200125.

### 2.5. Zebrafish Experimental Group

The young fish (three days post-fertilization, normal development) were randomly divided into 6 groups: Control, blank control group; Model, H_2_O_2_ (300 μM); Positive control, NAC (30 μM) + H_2_O_2_ (300 μM); C, C (25 μM) + H_2_O_2_ (300 μM); EC, EC (25 μM) + H_2_O_2_ (300 μM); ECG, ECG (25 μM) + H_2_O_2_ (300 μM); B1, B1 (25 μM) + H_2_O_2_ (300 μM); B2, B2 (25 μM) + H_2_O_2_ (300 μM); B3, B3 (25 μM) + H_2_O_2_ (300 μM); B4, B4 (25 μM) + H_2_O_2_ (300 μM); B1-G, B1-G (25 μM) + H_2_O_2_ (300 μM); B2-G, B2-G (25 μM) + H_2_O_2_ (300 μM); C1, C1 (25 μM) + H_2_O_2_ (300 μM) and treated for 4 days.

### 2.6. ROS Measurement

PC12 cells or zebrafish were exposed to 10 μM 2′,7′-Dichlorofluorescin diacetate solution in dark conditions for 30 min; the dye solution was then removed, and cells or zebrafish were washed with phosphate-buffered saline three times. Images were obtained using an Olympus laser scanning confocal microscope.

### 2.7. Assessment of MDA, GSH-Px, SOD, and CAT

The activities of MDA, GSH-Px, SOD, and CAT in PC12 cells or zebrafish were measured using the assay kits following the manufacturers’ protocols.

### 2.8. Nrf2 siRNA Transfection

PC12 cells were inoculated into 6-well plates (2 × 10^5^/well). After the cells adhered to the plate, they were washed with serum-free and antibody free Opti MEM cell culture medium three times, and Opti MEM culture medium was added to each well. siRNA was prepared to a final concentration of 80 nM, diluted with Lipofectamine 2000, mixed well, and left to stand for 10 min. The transfection complex was added to the cells and incubated for 6 h. The transfected cells were treated with C, EC, ECG, B1, B2, B3, B4, B1-G, B2-G, or C1 for 24 h and then with H_2_O_2_ (200 μM) for 24 h. Then, cell survival was detected.

### 2.9. Western Blotting

Protein samples were resolved by SDS-PAGE and transferred to polyvinylidene difuoride (PVDF) membranes. The blots were exposed to appropriate primary antibodies: Nrf2, HO-1, NQO1, Lamin B, GAPDH, and peroxidase-conjugated secondary antibodies. Protein bands were visualized using ECL plus Western blotting detection reagents.

### 2.10. Behavioral Observation of Zebrafish

A young fish was placed in each well of a 24 well plate and allowed to adapt for 1 h. Then, the movement of each fish was recorded within 10 min and its movement track and distance were analyzed.

### 2.11. Total RNA Extraction, Reverse Transcription, and Quantitative Real-Time Polymerase Chain Reaction

RNA was extracted using RNAiso following the manufacturer’s instructions. RNA was reverse transcribed using a PrimeScript RT Kit with gDNA eraser following the manufacturer’s instructions. Quantitative real-time PCR was performed with an Applied Biosystems Vii-7 Real-Time PCR system using SYBR Premix Ex Taq II. β-actin was used as a reference gene, and relative gene expression was calculated using the 2^−∆∆Ct^ method. The primer sequences utilized are listed in Table 1.

### 2.12. Statistical Analysis

The experimental results were statistically analyzed using one-way analysis of variance (ANOVA) and Duncan’s multiple range test; results are presented as means ± standard deviation (SD). A *p* < 0.05 was considered statistically significant. SPSS (version 20.0) was used for statistical analysis.

## 3. Results

### 3.1. Cytotoxicity of Procyanidins with Different Structures in PC12 Cells

**Figure 1:**
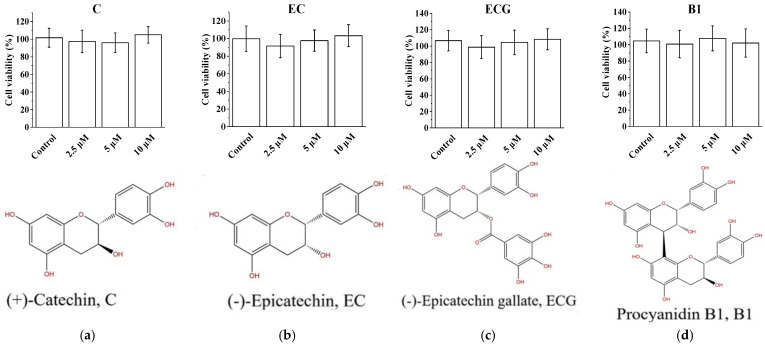

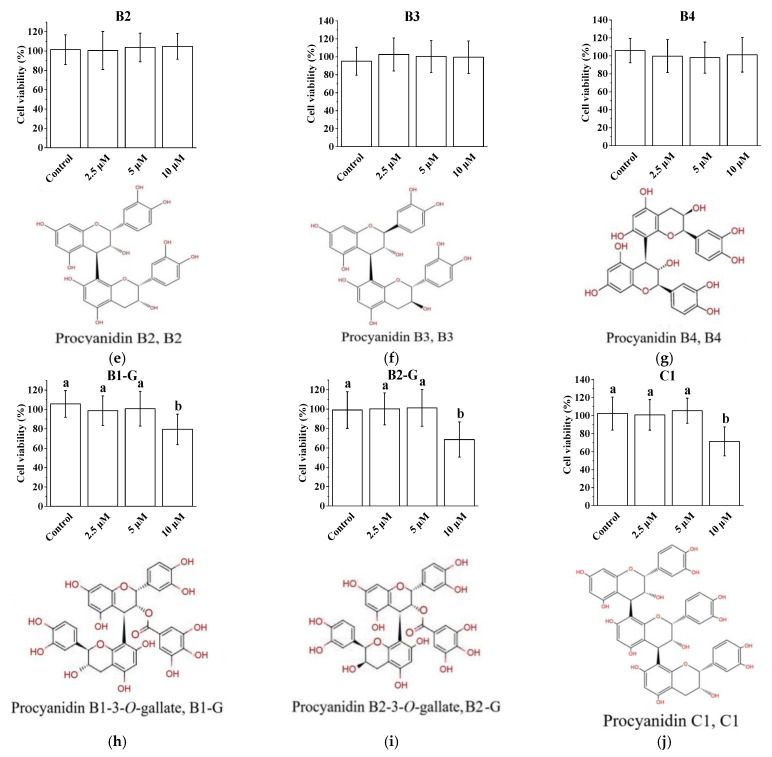
Cytotoxicity of different procyanidins in PC12 cells. (**a**) Survival of PC12 cells in the C treatment group; (**b**) Survival rate of PC12 cells in the EC treatment group; (**c**) Survival rate of PC12 cells in the ECG treatment group; (**d**) Survival rate of PC12 cells in the B1 treatment group; (**e**) Survival rate of PC12 cells in the B2 treatment group; (**f**) Survival rate of PC12 cells in the B3 treatment group; (**g**) Survival rate of PC12 cells in the B4 treatment group; (**h**) Survival rate of PC12 cells in the B1-G treatment group; (**i**) Survival rate of PC12 cells in the B2-G treatment group; (**j**) Survival rate of PC12 cells in the C1 treatment group. Data are expressed as the mean ± SD. All experiments were conducted six times. Values with different letters above each bar represent significant differences (*p* < 0.05, one-way ANOVA). PC12, rat pheochromocytoma cell line; EC, epicatechin; ECG, epicatechin gallate; B1, procyanidin B1; B2, procyanidin B2; B3, procyanidin B3; B4, procyanidin B4; B1-G, procyanidin B1-3-*O*-gallate; B2-G: procyanidin B2-3-*O*-gallate; C1: procyanidin C1; SD, standard deviation; ANOVA, one-way analysis of variance.

Ten procyanidins purified from grape seeds, including C, EC, and ECG; B1, B2, B3, B4, B1-G, and B2-G; and C1 (Figure 1), were selected for this study. Compared with the blank control group, procyanidins (C, EC, ECG, B1, B2, B3, B4, B1-G, B2-G and C1) at 2.5 μM and 5 μM had no significant effect on the survival of PC12 cells (Figure 1, *p* > 0.05). Procyanidins (C, EC, ECG, B1, B2, B3 and B4) at 10 μM also had no significant effect on the survival of PC12 cells (Figure 1, *p* > 0.05). However, the survival of PC12 cells treated with 10 μM B1-G, B2-G, and C1 was significantly lower than the blank control group (Figure 1, *p* < 0.05). Therefore, 2.5 μM and 5 μM of C, EC, ECG, B1, B2, B3, B4, B1-G, B2-G, and C1 were selected for further study.

### 3.2. Effects of Procyanidins with Different Structures on H_2_O_2_-Induced Damage in PC12 Cells

**Figure 2:**
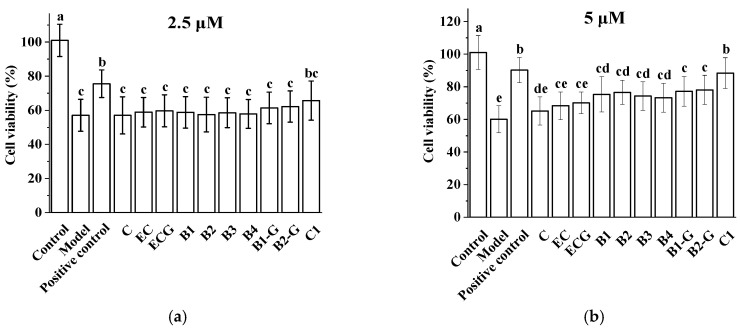
Effect of different procyanidins on the survival of PC12 cells induced by H_2_O_2_. (**a**) Effect of 2.5 μM procyanidins on the survival of PC12 cells induced by H_2_O_2_; (**b**) Effect of 5 μM procyanidins on the survival of PC12 cells induced by H_2_O_2_. Control, Blank control group; Model. H_2_O_2_ (200 μM); Positive control, NAC (20 μM) + H_2_O_2_ (200 μM); C, C + H_2_O_2_ (200 μM); EC, EC + H_2_O_2_ (200 μM); ECG, ECG + H_2_O_2_ (200 μM); B1, B1 + H_2_O_2_ (200 μM); B2, B2 + H_2_O_2_ (200 μM); B3, B3 + H_2_O_2_ (200 μM); B4, B4 + H_2_O_2_ (200 μM); B1-G, B1-G + H_2_O_2_ (200 μM); B2-G, B2-G + H_2_O_2_ (200 μM); C1, C1 + H_2_O_2_ (200 μM). Data are expressed as the mean ± SD. All experiments were conducted six times. Values with different letters above each bar represent significant differences (*p* < 0.05, one-way ANOVA). NAC, *N*-acetyl-l-cysteine.

The effects of grape seed procyanidins with different structures on the survival of PC12 cells induced by H_2_O_2_ were studied. Compared with the model group, 2.5 μM procyanidins (C, EC, ECG, B1, B2, B3, B4, B1-G, B2-G, and C1) had no significant protective effect on H_2_O_2_-induced PC12 cell injury (Figure 2a, *p* > 0.05), whereas 5 μM procyanidins (B1, B2, B3, B4, B1-G, B2-G, and C1) had significant protective effects on PC12 cell injury induced by H_2_O_2_ (Figure 2b, *p* < 0.05).

### 3.3. Effects of Procyanidins with Different Structures on Oxidative Stress in PC12 Cells Treated with H_2_O_2_

**Figure 3:**
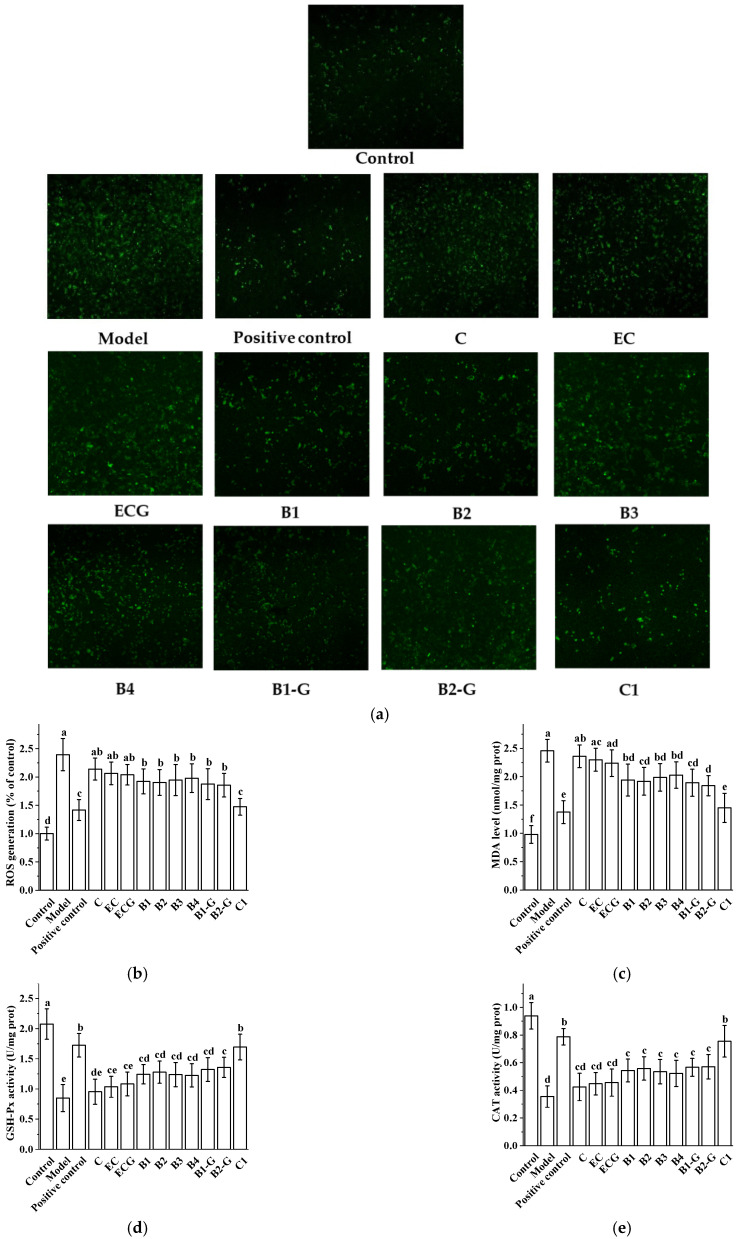

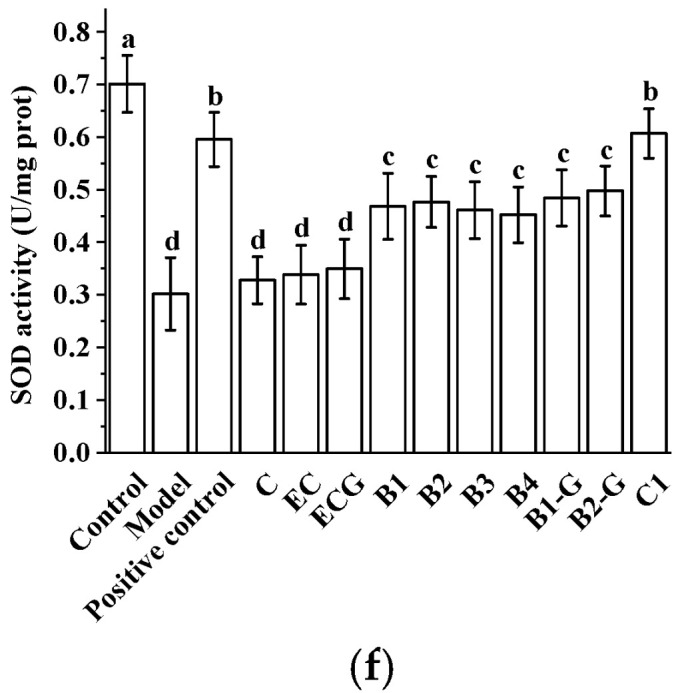
Effects of procyanidins with different structures on oxidative stress in PC12 cells treated with H_2_O_2_. (**a**) Representative fluorescence photomicrographs of PC12 cells; (**b**) ROS levels; (**c**) MDA levels; (**d**) GSH-Px activity; (**e**) CAT activity; (**f**) SOD activity. Data are expressed as the mean ± SD. Control, Blank control group; Model, H_2_O_2_ (200 μM); Positive control, NAC (20 μM) + H_2_O_2_ (200 μM); C, C (5 μM) + H_2_O_2_ (200 μM); EC, EC (5 μM) + H_2_O_2_ (200 μM); ECG, ECG (5 μM) + H_2_O_2_ (200 μM); B1, B1 (5 μM) + H_2_O_2_ (200 μM); B2, B2 (5 μM) + H_2_O_2_ (200 μM); B3, B3 (5 μM) + H_2_O_2_ (200 μM); B4, B4 (5 μM) + H_2_O_2_ (200 μM); B1-G, B1-G (5 μM) + H_2_O_2_ (200 μM); B2-G, B2-G (5 μM) + H_2_O_2_ (200 μM); C1, C1 (5 μM) + H_2_O_2_ (200 μM). All experiments were conducted three times. Values with different letters are significantly different (*p* < 0.05, one-way ANOVA). ROS, reactive oxygen species; MDA, malondialdehyde; GSH-Px, glutathione peroxidase; CAT, catalase; SOD, superoxide dismutase.

ROS and MDA content in cells is an index that directly reflects the level of oxidative stress [29,30,31]. GSH-Px, CAT, and SOD are important components of the antioxidant system, which can antagonize and block free radicals [32,33]. The higher the activity of GSH-Px, CAT, and SOD, the stronger the ability to scavenge oxygen free radicals [34]. Therefore, the antioxidant capacity of cells can be reflected by detecting the activities of GSH-Px, CAT, and SOD. Compared with the model group, treatment with different procyanidins inhibited H_2_O_2_-induced ROS and MDA levels and increased the activities of GSH-Px, CAT, and SOD in PC12 cells. The treatment effect of the procyanidin trimer C1 was greater than that of the procyanidin dimers (B1, B2, B3, B4, B1-G, and B2-G) (Figure 3, *p* < 0.05). The effect in the procyanidin monomers (C, EC, and ECG) treatment group was less than in the procyanidin dimers (B1, B2, B3, B4, B1-G, and B2-G) and procyanidin trimer C1 treatment groups (Figure 3).

### 3.4. Effects of Procyanidins with Different Structures on the Nuclear Factor-Erythroid 2-Related Factor 2 (Nrf2)/Antioxidant Response Element (ARE) Pathway in PC12 Cells Treated with H_2_O_2_

**Figure 4:**
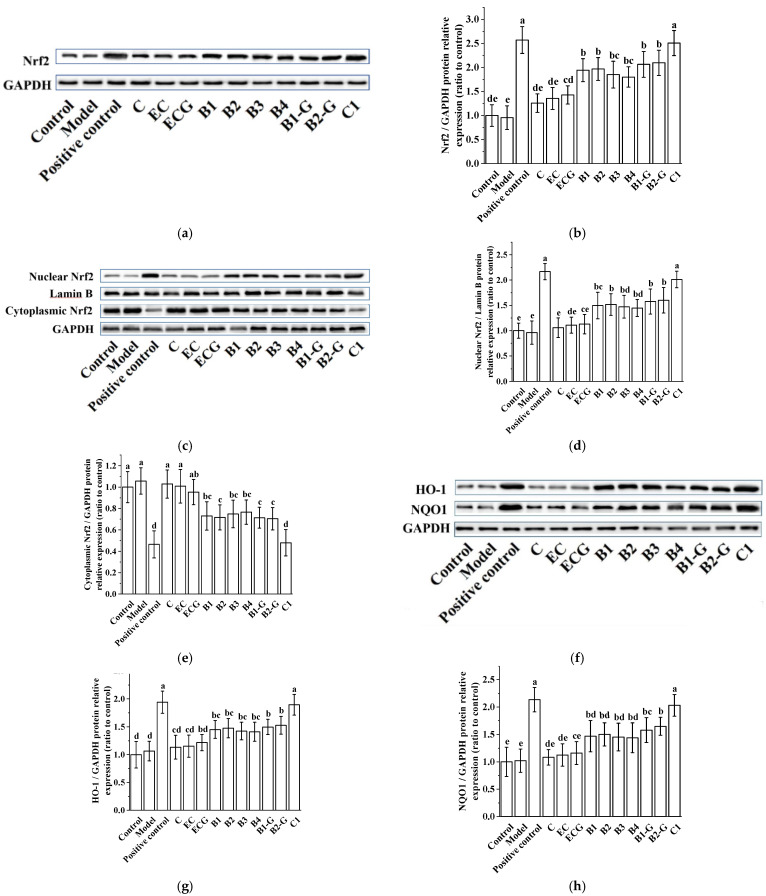
Effects of procyanidins with different structures on the Nrf2/ARE pathway in PC12 cells treated with H_2_O_2_. (**a**) Protein levels of Nrf2, as determined by Western blotting; (**b**) Relative Nrf2/GAPDH protein expression (ratio to control); (**c**) Protein expression levels of nuclear Nrf2 and cytoplasmic Nrf2, as determined by Western blotting; (**d**) Relative nuclear Nrf2/Lamin B protein expression (ratio to control); (**e**) Relative cytoplasmic Nrf2/GAPDH protein expression (ratio to control); (**f**) Protein levels of HO-1 and NQO1, as determined by Western blotting; (**g**) Relative HO-1/GAPDH protein expression (ratio to control); (**h**) Relative NQO1/GAPDH protein expression (ratio to control). Data are expressed as the mean ± SD. Control, Blank control group; Model, H_2_O_2_ (200 μM); Positive control, NAC (20 μM) + H_2_O_2_ (200 μM); C, C (5 μM) + H_2_O_2_ (200 μM); EC, EC (5 μM) + H_2_O_2_ (200 μM); ECG, ECG (5 μM) + H_2_O_2_ (200 μM); B1, B1 (5 μM) + H_2_O_2_ (200 μM); B2, B2 (5 μM) + H_2_O_2_ (200 μM); B3, B3 (5 μM) + H_2_O_2_ (200 μM); B4, B4 (5 μM) + H_2_O_2_ (200 μM); B1-G, B1-G (5 μM) + H_2_O_2_ (200 μM); B2-G, B2-G (5 μM) + H_2_O_2_ (200 μM); C1, C1 (5 μM) + H_2_O_2_ (200 μM). All experiments were conducted three times. Values with different letters are significantly different (*p* < 0.05, one-way ANOVA). Nrf2, nuclear factor-erythroid 2-related factor 2; ARE, antioxidant response element; HO-1, heme oxygenase 1; NQO1, NAD(P)H: quinone oxidoreductase 1.

The different structures of the procyanidin treatments may up-regulate the expression of the Nrf2 protein, leading to transfer from the cytoplasm to the nucleus and accumulation in the nucleus, thereby up-regulating the expression of NAD(P)H: quinone oxidoreductase 1 (NQO1) and heme oxygenase 1 (HO-1) in H_2_O_2_ injured PC12 cells. Our results showed significant differences between the procyanidin dimers (B1, B2, B3, B4, B1-G, and B2-G) and procyanidin trimer C1 treatment groups compared to the model group (Figure 4, *p* < 0.05).

### 3.5. Verification of the Effect of Nrf2 in the Protection of Cells Treated with Procyanidins

**Figure 5:**
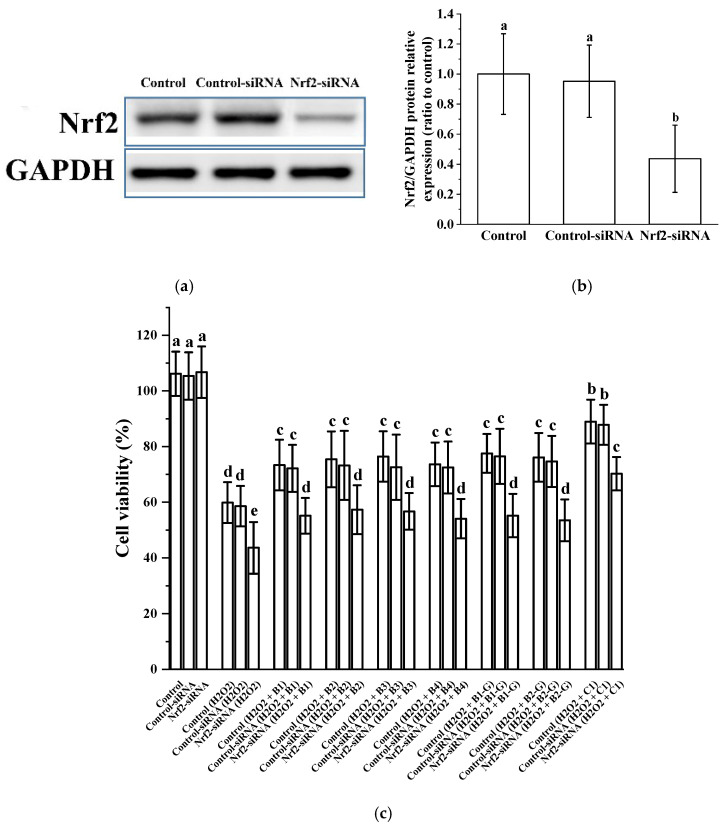
Verification of the role of Nrf2 in the protective effect of procyanidins with different structures. (**a**) Knockout efficiency was confirmed by determination of Nrf2 protein expression using Western blotting; (**b**) Relative Nrf2/GAPDH protein expression (ratio to control); (**c**) Cell viability. Data are shown as the mean ± SD. All experiments were conducted three times. Values with different letters are significantly different (*p* < 0.05, one-way ANOVA).

We further evaluated the role of Nrf2/ARE pathway activation in procyanidin-mediated neuroprotection. Results showed that the expression of Nrf2 in PC12 cells transfected with Nrf2 siRNA decreased significantly, blocking the protective effect of procyanidins in PC12 cells challenged with H_2_O_2_ (Figure 5). These results suggest that procyanidins (B1, B2, B3, B4, B1-G, B2-G, and C1) may protect neurons from oxidative stress by activating the Nrf2/ARE pathway.

### 3.6. Effects of Procyanidins with Different Structures on Exercise Capacity in Zebrafish Treated with H_2_O_2_

**Figure 6:**
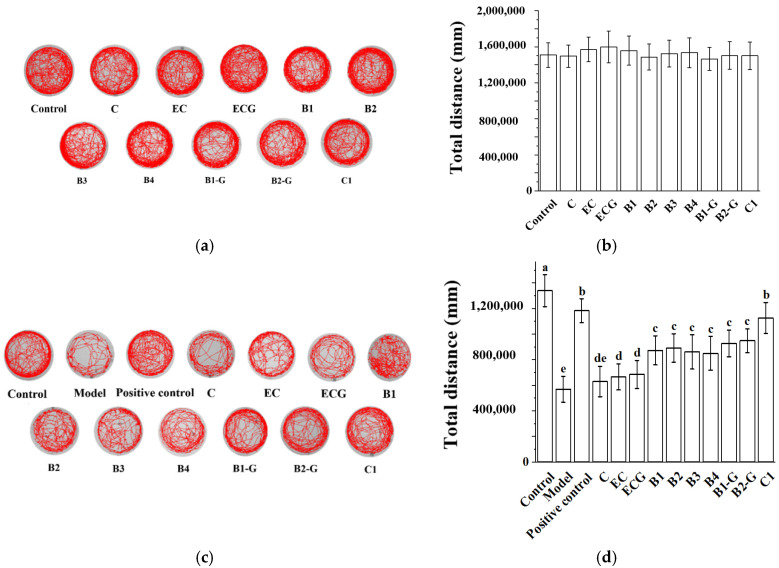
Effects of procyanidins with different structures on exercise ability in zebrafish treated with H_2_O_2_. (**a**) Swimming traces of zebrafish in each group; (**b**) Average total distance of zebrafish in each group; (**c**) Swimming traces of zebrafish in each group; (**d**) Average total distance of zebrafish in each group. Data are expressed as the mean ± SD. Control, Blank control group; Model, H_2_O_2_ (300 μM); Positive control, NAC (30 μM) + H_2_O_2_ (300 μM); C, C (25 μM) + H_2_O_2_ (300 μM); EC, EC (25 μM) + H_2_O_2_ (300 μM); ECG, ECG (25 μM) + H_2_O_2_ (300 μM); B1, B1 (25 μM) + H_2_O_2_ (300 μM); B2, B2 (25 μM) + H_2_O_2_ (300 μM); B3, B3 (25 μM) + H_2_O_2_ (300 μM); B4, B4 (25 μM) + H_2_O_2_ (300 μM); B1-G, B1-G (25 μM) + H_2_O_2_ (300 μM); B2-G, B2-G (25 μM) + H_2_O_2_ (300 μM); C1, C1 (25 μM) + H_2_O_2_ (300 μM). All experiments were conducted three times. Values with different letters are significantly different (*p* < 0.05, one-way ANOVA).

Neurodegenerative diseases often exhibit symptoms of motor behavior disorders [35,36,37]. Therefore, this experiment studied the effects of different structures of procyanidins on exercise capacity of zebrafish treated with H_2_O_2_. Overall, 25 μM of procyanidins (C, EC, ECG, B1, B2, B3, B4, B1-G, B2-G, C1) did not decrease the exercise capacity of zebrafish (Figure 6a,b) and had a protective effect on zebrafish damaged by H_2_O_2_ (Figure 6 c,d). The protective effect of the procyanidin trimer C1 was better than that of the procyanidin dimers (B1, B2, B3, B4, B1-G, B2-G) (Figure 6c,d, *p* < 0.05). There was no significant difference between the procyanidin monomer (C, EC, ECG) treatment groups and the model group (Figure 6c,d, *p* > 0.05).

### 3.7. Effects of Procyanidins with Different Structures on Oxidative Stress in Zebrafish Treated with H_2_O_2_

**Figure 7:**
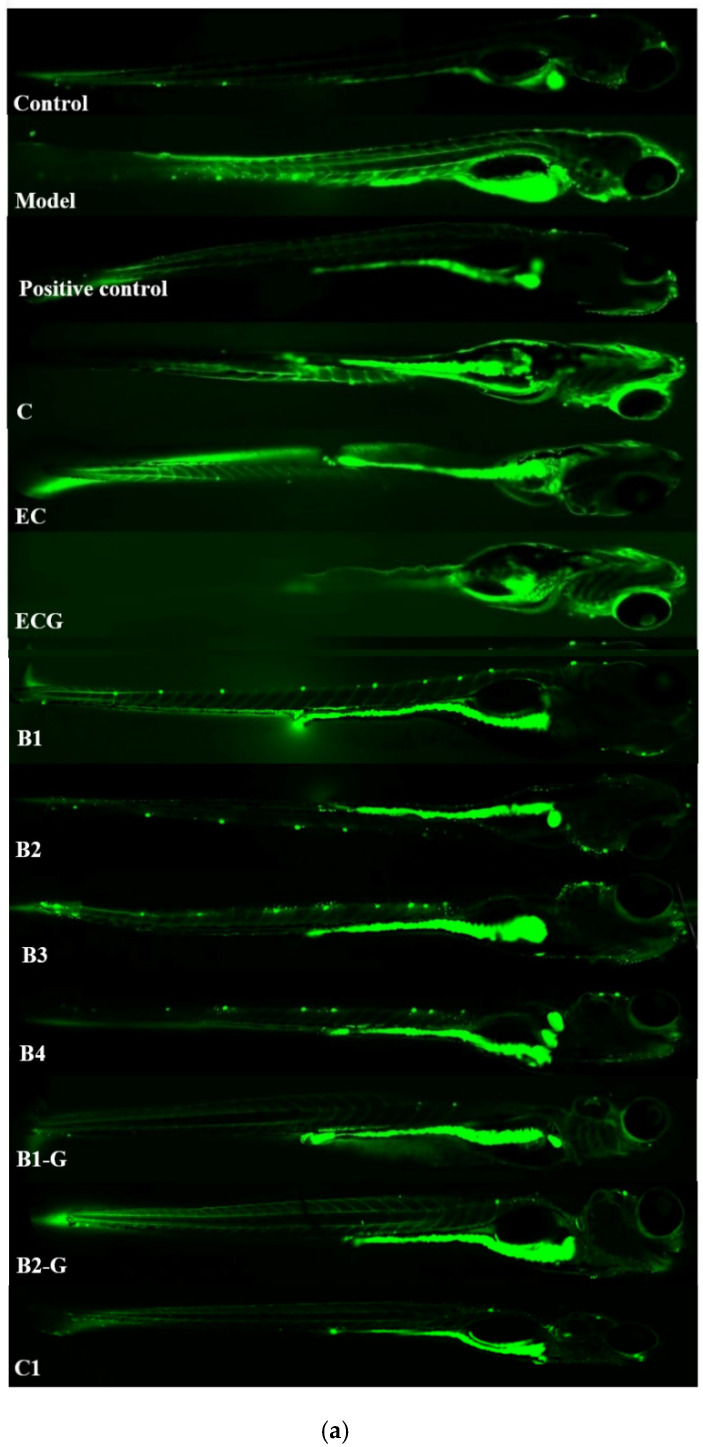

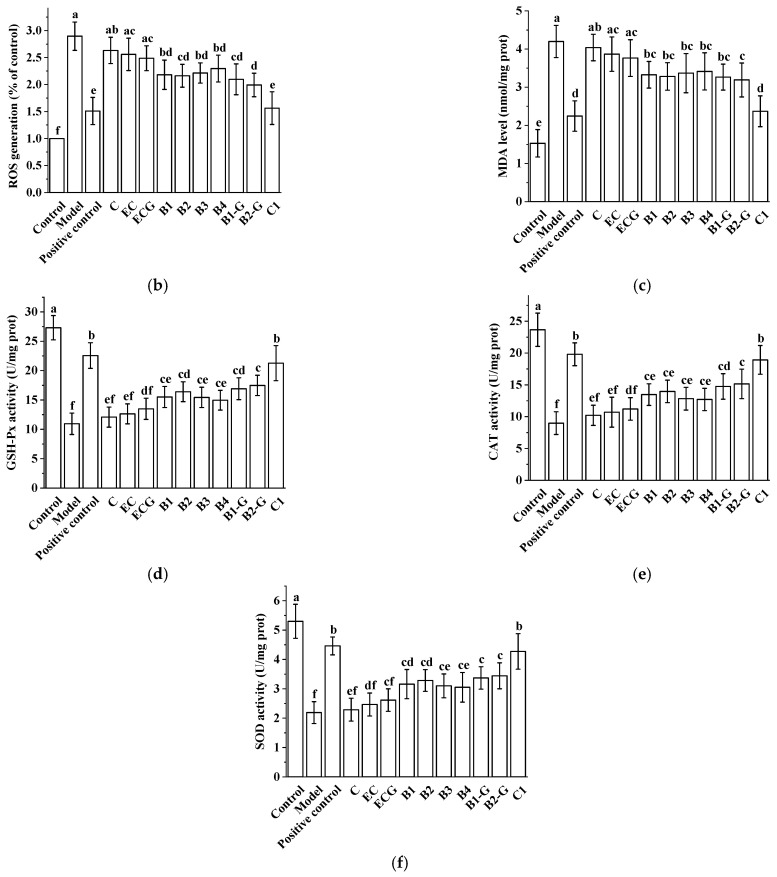
Effects of procyanidins with different structures on oxidative stress in zebrafish treated with H_2_O_2_. (**a**) Representative fluorescence photomicrographs of zebrafish; (**b**) ROS levels; (**c**) MDA levels; (**d**) GSH-Px activity; (**e**) CAT activity; (**f**) SOD activity. Data are expressed as the mean ± SD. Control, Blank control group; Model, H_2_O_2_ (300 μM); Positive control, NAC (30 μM) + H_2_O_2_ (300 μM); C, C (25 μM) + H_2_O_2_ (300 μM); EC, EC (25 μM) + H_2_O_2_ (300 μM); ECG, ECG (25 μM) + H_2_O_2_ (300 μM); B1, B1 (25 μM) + H_2_O_2_ (300 μM); B2, B2 (25 μM) + H_2_O_2_ (300 μM); B3, B3 (25 μM) + H_2_O_2_ (300 μM); B4, B4 (25 μM) + H_2_O_2_ (300 μM); B1-G, B1-G (25 μM) + H_2_O_2_ (300 μM); B2-G, B2-G (25 μM) + H_2_O_2_ (300 μM); C1, C1 (25 μM) + H_2_O_2_ (300 μM). All experiments were conducted three times. Values with different letters are significantly different (*p* < 0.05, one-way ANOVA).

The contents of ROS and MDA in different procyanidin treatment groups were lower than the model group, and the activities of antioxidant enzymes (GSH-Px, CAT, and SOD) were higher than the model group. There was no significant difference between the procyanidin monomer (C, EC, ECG) treatment groups and the model group (Figure 7, *p* > 0.05); however, there was a significant difference between the procyanidin dimer (B1, B2, B3, B4, B1-G, B2-G) treatment groups and the procyanidin trimer C1 treatment group and the model group (Figure 7, *p* < 0.05). The effect of the procyanidin trimer C1 treatment group on reducing the content of ROS and MDA and increasing the activity of antioxidant enzymes (GSH-Px, CAT, and SOD) was greater than that of the other treatment groups (Figure 7).

### 3.8. Effects of Procyanidins with Different Structures on the Nrf2/ARE Pathway in Zebrafish Treated with H_2_O_2_

**Figure 8:**
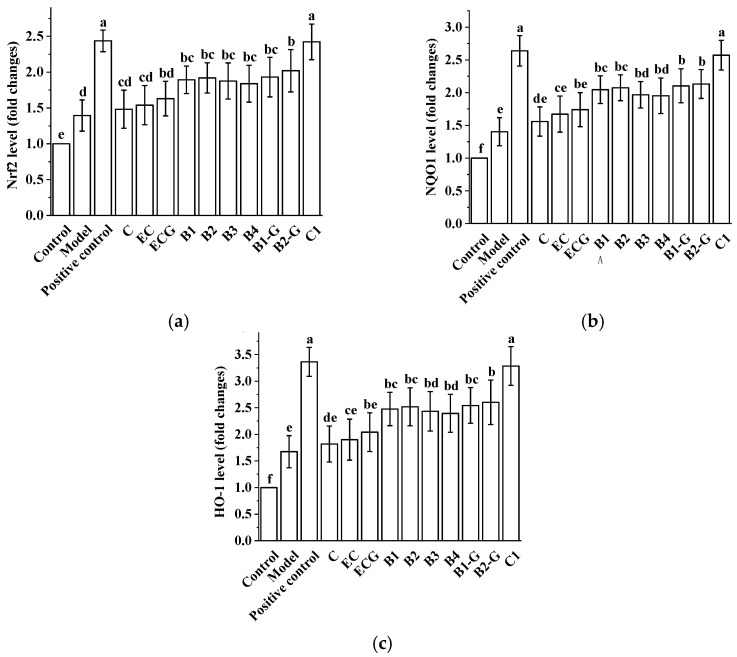
Effects of procyanidins with different structures on the Nrf2/ARE pathway in zebrafish treated with H_2_O_2_. (**a**) Nrf2 levels; (**b**) NQO1 levels; (**c**) HO-1 levels. Data are expressed as the mean ± SD. Control, Blank control group; Model, H_2_O_2_ (300 μM); Positive control, NAC (30 μM) + H_2_O_2_ (300 μM); C, C (25 μM) + H_2_O_2_ (300 μM); EC, EC (25 μM) + H_2_O_2_ (300 μM); ECG, ECG (25 μM) + H_2_O_2_ (300 μM); B1, B1 (25 μM) + H_2_O_2_ (300 μM); B2, B2 (25 μM) + H_2_O_2_ (300 μM); B3, B3 (25 μM) + H_2_O_2_ (300 μM); B4, B4 (25 μM) + H_2_O_2_ (300 μM); B1-G, B1-G (25 μM) + H_2_O_2_ (300 μM); B2-G, B2-G (25 μM) + H_2_O_2_ (300 μM); C1, C1 (25 μM) + H_2_O_2_ (300 μM). All experiments were conducted three times. Values with different letters are significantly different (*p* < 0.05, one-way ANOVA).

Different procyanidin treatments can up-regulate the expression of Nrf2 and the related genes NQO1 and HO-1, which are downstream of the Nrf2/ARE pathway in H_2_O_2_-induced zebrafish. There was no significant difference between the procyanidin monomer (C, EC, ECG) treatment groups and the model group (Figure 8, *p* > 0.05); however, there was a significant difference between the procyanidin dimer (B1, B2, B3, B4, B1-G, B2-G) treatment groups and the procyanidin trimer C1 treatment group and the model group (Figure 8, *p* < 0.05). Up-regulation of the Nrf2, NQO1, and HO-1 genes in the procyanidin trimer C1 treatment group was greater than in the other treatment groups.

## 4. Discussion

Excessive production of ROS and oxidative damage caused by a damaged antioxidant defense system plays a key role in neurodegenerative diseases. Antioxidant supplementation may help to prevent or alleviate neurodegenerative diseases [4,5,6]. Procyanidin is a natural antioxidant with a stronger antioxidant capacity than vitamins C and E [7,8,9]. The bioavailability of procyanidins is affected by their structure, and different plants contain procyanidins with distinct structural types. Due to the large number of procyanidins, complex separation conditions, and difficulty with purification, previous studies have mainly focused on mixtures of procyanidins or individual procyanidins, and less on the structure–activity relationship of a variety of procyanidins [15,16]. Previous studies have found that procyanidins can play a neuroprotective role by regulating the Nrf2/ARE pathway, but the relationship between the neuroprotective effect of procyanidins and the structural characteristics of procyanidins is not clear [10]. Grape seed is one of the most abundant sources of procyanidins in nature [13]. Most procyanidin products are from grape seed [14]. H_2_O_2_ can penetrate the cell membrane and cause oxidative stress. This method can thus be used to establish an oxidative stress model. Therefore, 10 procyanidins from grape seeds were selected for study of the relationship between the neuroprotective effects of procyanidins and their structural characteristics using H_2_O_2_-damaged PC12 cells and zebrafish modeling.

This study evaluated the effects of 10 grape seed-derived procyanidins on the survival of PC12 cells and the motor capacity of zebrafish challenged with H_2_O_2_. The results showed that treatment with 5 μM procyanidins resulted in a protective effect on the survival of PC12 cells, and treatment with 25 μM procyanidins resulted in a protective effect on the motor capacity of zebrafish challenged with H_2_O_2_. There was no significant difference between procyanidin monomers (C, EC, ECG) and procyanidin dimers (B1, B2, B3, B4, B1-G, B2-G). Additionally, there was no significant difference between the procyanidin monomer (C, EC, ECG) treatment groups and the model group (*p* > 0.05). However, there was a significant difference between the procyanidin dimer (B1, B2, B3, B4, B1-G, B2-G) and the procyanidin trimer C1 treatment groups and the model group (*p* < 0.05). The protective effect of procyanidin trimer C1 treatment on the survival rate of PC12 cells and the motor capacity of zebrafish challenged with H_2_O_2_ was greater than that of the other treatment groups. This shows that the protective effect of procyanidins on the viability of PC12 cells damaged by H_2_O_2_, and the exercise ability of zebrafish damaged by H_2_O_2_, were positively correlated with the degree of procyanidin polymerization.

ROS is an indicator that directly reflects oxidative stress levels [29,30]. MDA is the end product of the peroxidation reaction of free radicals acting on membrane lipids under the catalysis of metal ions, which reflects the degree of oxidative damage [31]. We evaluated the effects of different procyanidins from grape seeds on the content of ROS and MDA in PC12 cells and zebrafish challenged with H_2_O_2_. Compared with the model group, procyanidin dimers (B1, B2, B3, B4, B1-G, B2-G) and the procyanidin trimer C1 significantly inhibited H_2_O_2_-induced increases in ROS and MDA in PC12 cells and zebrafish (*p* < 0.05). GSH-Px, CAT, and SOD are important components of the antioxidant system, which can antagonize and block free radicals [32,33]. The higher the activity of GSH-Px, CAT, and SOD, the stronger the ability to scavenge oxygen free radicals [34]. Therefore, the detection of GSH-Px, CAT, and SOD activity can reflect antioxidant capacity. Results showed that compared with the model group, procyanidin dimers (B1, B2, B3, B4, B1-G, B2-G) and the procyanidin trimer C1 increased the activity of GSH-Px, CAT, and SOD in PC12 cells and zebrafish treated with H_2_O_2_ (*p* < 0.05). Procyanidin monomers (C, EC, ECG) also exhibited a reversal effect on the decrease of GSH-Px, CAT, and SOD activities in PC12 cells and zebrafish treated with H_2_O_2_; however, there was no significant difference compared to the model group (*p* > 0.05). This shows that the ability of the procyanidin trimer to reduce ROS and MDA contents and increase the activity of GSH-Px, CAT, and SOD was better than that of the procyanidin dimers (B1, B2, B3, B4, B1-G, B2-G) and procyanidin monomers (C, EC, ECG).

The Nrf2/ARE pathway is an important antioxidant pathway [38]. Under normal conditions, Nrf2 binds to the inhibitory protein Keap1 in the cytoplasm [39]. When the cysteine residue at the terminus of Keap1 is damaged by oxidation, a redox reaction occurs and the conformation of Keap1 changes, causing it to separate from Nrf2. Once Nrf2 is activated, it moves from the cytoplasm to the nucleus, inducing the transcription of ARE-dependent antioxidant genes and enhancing antioxidant capacity [40,41,42,43,44,45]. Our results showed that after treatment with different procyanidins from grape seeds, expression of the Nrf2 gene was up-regulated in zebrafish. In PC12 cells, the expression of total Nrf2 protein increased, Nrf2 protein in the cytoplasm gradually decreased, and Nrf2 protein in the nucleus gradually increased. There were significant differences between the model group and the procyanidin dimer (B1, B2, B3, B4, B1-G, B2-G) and procyanidin trimer C1 groups. The presence of Nrf2 in the nucleus promotes its binding to ARE, catalyzing transcription and inducing the expression of the phase II detoxification enzyme genes NQO1 and HO-1. NQO1 is an antioxidant enzyme that uses NADH or NADPH as a reduction cofactor to participate in the two-electron reduction of endogenous quinones [46]. Chaperone HO-1 cooperates with cytochrome p450 to catalyze the degradation of heme to biliverdin, which is then converted to bilirubin. Both biliverdin and bilirubin exhibit antioxidant and immunomodulatory properties [47,48]. Our results showed that grape seed-derived procyanidin dimers (B1, B2, B3, B4, B1-G, B2-G) and procyanidin trimer C1 treatment in PC12 cells and zebrafish reversed the down-regulation of HO-1 and NQO1 expression induced by H_2_O_2_. Nrf2 siRNA treatment was used to further evaluate the role of Nrf2/ARE pathway activation in the procyanidin dimer (B1, B2, B3, B4, B1-G, B2-G) and procyanidin trimer C1 treatment groups. Results showed that the expression of Nrf2 in PC12 cells transfected with Nrf2 siRNA decreased significantly, and the protective effect of the procyanidin dimer (B1, B2, B3, B4, B1-G, B2-G) and procyanidin trimer C1 treatments in PC12 cells treated with H_2_O_2_ was blocked. This shows that procyanidins activated the Nrf2/ARE pathway, causing transfer of Nrf2 from the cytoplasm to the nucleus where it accumulated, and increased expression of the phase II detoxification enzymes HO-1 and NQO1, which are downstream of the Nrf2/ARE pathway. The regulatory effect of procyanidins on the Nrf2/ARE pathway was positively correlated with the degree of procyanidin polymerization.

## 5. Conclusions

Procyanidins from grape seeds protected PC12 cells from H_2_O_2_ induced decreases in cell viability, improved the movement behavior disorder of zebrafish caused by H_2_O_2_, reversed H_2_O_2_-induced increases in ROS and MDA and decreases in antioxidant enzyme (GSH-Px, CAT and SOD) activity, and regulated the Nrf2/ARE pathway. The neuroprotective effect in the procyanidin trimer C1 treatment group was greater than in the procyanidin dimer (B1, B2, B3, B4, B1-G, B2-G) treatment groups, and the neuroprotective effect of the procyanidin monomer (C, EC, ECG) treatment group was less than that of the procyanidin trimer C1 and procyanidin dimer (B1, B2, B3, B4, B1-G, B2-G) treatment groups. Procyanidins play a neuroprotective role through activation of the Nrf2/ARE pathway and its downstream detoxification enzymes (HO-1, NQO1) and antioxidant enzymes (GSH-Px, CAT, and SOD). The degree of procyanidin polymerization is an important factor affecting the neuroprotective effect of procyanidins.

## Figures and Tables

**Table 1:** Sequences of primers for quantitative real-time PCR.

Genes	Forward Primer	Reverse Primer
β-Actin	CACTGAGGCTCCCCTGAATC	GGGTCACACCATCACCAGAG
Nrf2	CTGCTGTCACTCCCAGAGTT	GCCGTAGTTTTGGGTTGGTG
HO-1	AAGAGCTGGACAGAAACGCA	AGAAGTGCTCCAAGTCCTGC
NQO1	AAGCCTCTGTCCTTTGCTCC	TGCTGTGGTAATGCCGTAGG

Nrf2: nuclear factor-erythroid 2-related factor 2; HO-1: heme oxygenase 1; NQO1: quinone oxidoreductase 1.

## Data Availability

Data is contained within the article.
